# The role played by alternative splicing in antigenic variability in human endo-parasites

**DOI:** 10.1186/1756-3305-7-53

**Published:** 2014-01-28

**Authors:** Rodney Hull, Zodwa Dlamini

**Affiliations:** 1 University of South Africa, College of Agriculture and Environmental Sciences, College of Agriculture and Environmental Sciences, C/o Christiaan de Wet and Pioneer Avenue, Private Bag X6, Florida Science Campus, Florida, Johannesburg 1710, South Africa

**Keywords:** *Plasmodium*, *Schistosoma*, *Trypanosoma*, HIV, Alternate splicing, Immune evasion

## Abstract

Endo-parasites that affect humans include *Plasmodium*, the causative agent of malaria, which remains one of the leading causes of death in human beings. Despite decades of research, vaccines to this and other endo-parasites remain elusive. This is in part due to the hyper-variability of the parasites surface proteins. Generally these surface proteins are encoded by a large family of genes, with only one being dominantly expressed at certain life stages. Another layer of complexity can be introduced through the alternative splicing of these surface proteins. The resulting isoforms may differ from each other with regard to cell localisation, substrate affinities and functions. They may even differ in structure to the extent that they are no longer recognised by the host’s immune system. In many cases this leads to changes in the N terminus of these proteins. The geographical localisation of endo-parasitic infections around the tropics and the highest incidences of HIV-1 infection in the same areas, adds a further layer of complexity as parasitic infections affect the host immune system resulting in higher HIV infection rates, faster disease progression, and an increase in the severity of infections and complications in HIV diagnosis. This review discusses some examples of parasite surface proteins that are alternatively spliced in trypanosomes*, Plasmodium* and the parasitic worm *Schistosoma* as well as what role alternate splicing may play in the interaction between HIV and these endo-parasites.

## Review

### Introduction

**Figure 1:**
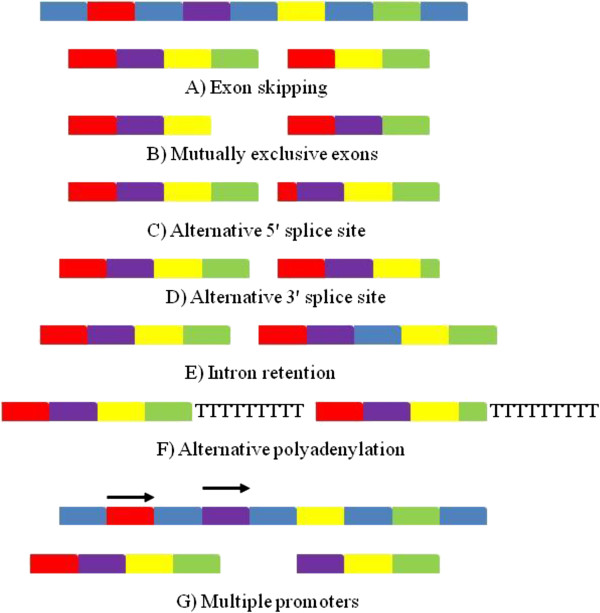
**Various forms of alternative splicing.** The introns are marked in blue with the exons marked in red, purple, yellow and green as the sit in order in the gene. **(A)** Exon skipping occurs when an exon is excluded. **(B)** Mutually exclusive exons implies that one exon is included while another is excised **(C and D)**. Alternative splice sites involve the 5’ and 3’ exons being shortened due to internal splice sites. **(E)** Intron retention occurs when an intron is retained and not excised. **(F)** Alternative polyadenylation sites can change the length of the 3’ untranslated region. **(G)** Alternate promoters can change transcription initiation sites.

The expression of genes can be controlled at many levels, these include transcriptional regulation, post-transcriptional regulation, translational regulation, mRNA degradation, protein degradation and through the actions of inhibitory proteins. One of the most important and complex mechanisms of post-transcriptional control involves alternate selection of splice sites within the pre-mRNA, which allows multiple different protein products with different functions to be coded for by a single gene [1]. There are five basic forms of alternative splicing (Figure 1). These are exon skipping, mutually exclusive exons, alternative 5’ or 3’ splice sites and intron retention. Two other forms of splice variants occur but they are not normally defined as alternate splicing. These are multiple promoters and multiple polyadenylation sites [2]. Exon splicing involves the removal or retention of an exon. Mutually exclusive exons occur when only one of a set of exons is retained the other is spliced out. Alternative 3’ and 5’ splice sites result in the sequence being extended or truncated at the 3’ or 5’ end due the occurrence of an alternate splicing site at either end. Intron retention occurs when an intron is included and not spliced out of the transcript. Alternative polyadenylation sites can shorten the transcript from its 3’ end. Finally, multiple promoters can lead to transcription starting either upstream or downstream of the first promoter creating a different transcript [2]. RNA splicing is carried out by the spliceosome, which is a large complex of proteins that include five small nuclear RNAs (U1, U2, U4, U5 and U6) and over 150 proteins. The association of these proteins with the snRNAs forms the snRNP (RNA protein complex). Seven of the associated proteins (SM proteins) form a ring around a conserved motif on the snRNA. The pre-mRNA contains short conserved sequences at the 5’ splice site, 3’ splice site and the branch site which is located upstream of the 3’ splice site. U1 associates with the 5’ splice site and U2 with the branch site [3]. The tri snRNP complex U4, U5 and U6 then associate with the pre-mRNA, U1 and U4 are ejected from the complex and the now activated spliceosome performs the first step where the branch site of the intron carries out a nucleophillic attack of the 5’ splice site. The result is the formation of a lariat structure as the 5’ end of the intron is bound to the branch site. The second step is then carried out as the 5’ end of the exon attacks the 3’ splice site resulting in the 5’ and 3’ exons joining. The lariat is then ejected along with U2, U5 and U6 [3].

In order to survive in the hostile environment presented by the host organism, parasites have evolved multiple mechanisms to evade the host’s immune system. Firstly, the parasites may hide in immune privileged tissue such as the central nervous system or fat tissue [4,5]. Secondly parasites can shield surface components to avoid recognition, which is commonly achieved by binding host molecules leading to the parasite being recognized as self by the host immune system [6-8]. Thirdly parasites may actively interfere with the hosts immune system by blocking signaling pathways required to activate different immune responses [9]. Finally they may change their antigenic surface molecules during infection. In order to do this many parasites have multiple surface variants within their genomes which are successively expressed [9]. Opportunistic infections by parasites in HIV patients are a common cause of mortality with most of these parasitic infections being due to protozoan and helminth parasites [10]. Of particular interest here are protozoan parasites of the genera *Plasmodium*, *Trypanosoma* and helminth parasites of the genus *Schistosoma.* The prevalence of these parasites varies geographically but collectively they are common in South America, Africa and South East Asia [10,11]. This coincides with the location of the highest prevalence of HIV infection [11,12].

This review will discuss the role that alternative splicing may play in the ability of a parasite to evade the immune system of the host. The discovery of alternate splicing giving rise to different isoforms of antigenic proteins seems to imply a further means of immune evasion by the parasite by avoiding immune detection. Some of these alternate splicing events give rise to isoforms with different functions allowing different interactions between the host and the parasite. Finally this review will examine the role played by alternative splicing in the interaction between HIV and parasites.

## Alternate splicing in protozoan parasites

### Trypanosomes

**Figure 2:**
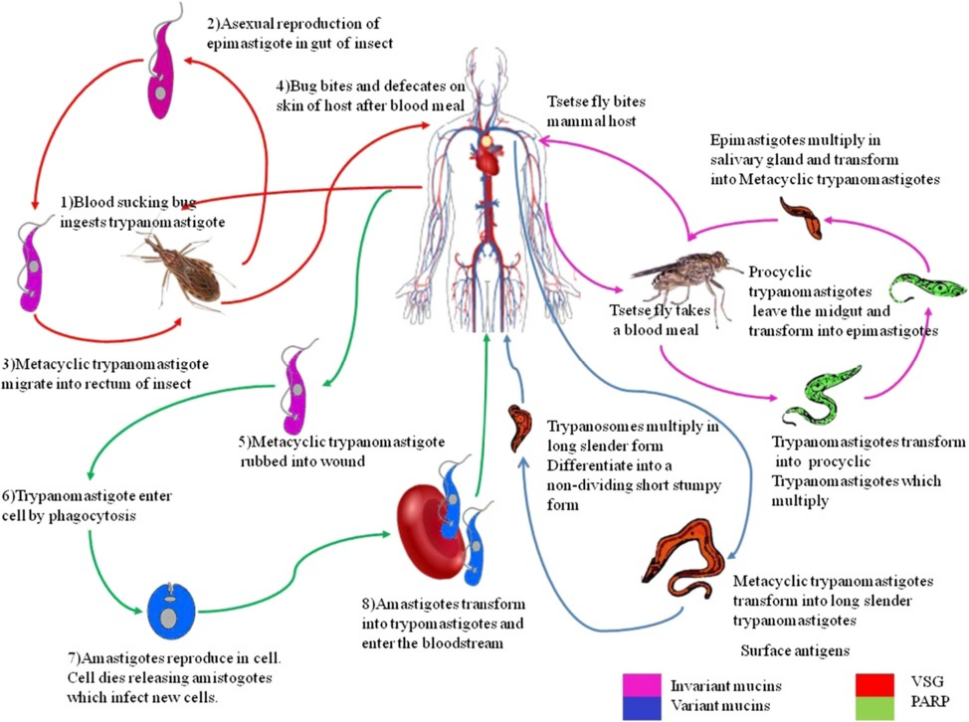
**The lifecycle of *Trypanosoma cruzi *and *Trypanosoma brucei.*** The life cycle of *T. cruzi* is shown on the left, that of *T. brucei* on the right. The two parasites use different strategies to escape the immune system of the mammalian host. *T. cruzi* enters cells to get away from the immune system. *T. brucei* escapes from the immune response by expressing a new VSG. These surface antigens vary after a period of time allowing the parasite to continually evade the host immune system. The parasites are coloured according to the surface antigens they display. Antigenic variation most likely evolved to aid in the transmission between the arthropod and mammalian hosts. Both pathogens express highly variant surface molecules Variant surface glycoprotiens (VSG) and variant mucins. However, in the arthropod host the parasites express invariant mucins and procyclic acidic repertoire protein (PARP).

*Trypanosoma* is a genus of unicellular, flagellate, protozoan parasites, which require more than one host to complete their lifecycle (Figure 2). They are generally transmitted by blood feeding invertebrates and have a vertebrate host. In order to penetrate the host’s cells *Trypanosoma* use multiple methods including active penetration, active induction of receptor-mediated phagocytosis, and opsonin-mediated phagocytosis. Following invasion the parasite causes cells to rupture releasing trypomastigotes which then multiply. Trypanosomes belong to the class Kinetoplastida and all members of this class possess a DNA containing granule located within their single mitochondrion [13].

*Trypanosoma brucei* is a uniquely African trypanosome parasite that causes African sleeping sickness. Nearly 60 million people are at risk from this disease. *T. brucei* also causes damage to livestock populations. The parasite is spread by the tsetse fly *Glossina moristans moristans.* The genome of *T. brucei* is 11 mega bases in size and contains approximately 9000 predicted genes with 900 pseudogenes. Approximately 1700 of these genes are unique to *T. brucei*[14]. Studies indicate that on average there are 2.6 splice sites for every gene in *T. brucei*. The number of genes with internal splice sites was estimated to be around 488 [15]. Analysis of alternative splicing sites in the genome of *T. brucei* pointed to the RNA splicing patterns changing in the different life stages of the parasite. It was suggested that this could be due to differences between the different life stages with regards to: the initiation of transcription, the rate of mRNA degradation or the recognition of splice sites [15]. It can be concluded that alternative splicing is required for the development of the parasite in the different host organisms.

*Trypanosoma cruzi* is the infectious agent that causes Chagas disease. This chronic illness is characterized by cardiomyopathy and the degeneration of the smooth muscles in the digestive tract. The surface of the parasite is covered in mucintype O-glycosylated sialoglycoproteins. These mucin type O-glycosylated proteins are predicted to play a role in host cell attachment. They also help to protect the parasite from T cell associated killing. These molecules are also antigenic and were found to contain alternate translation start codons preceded by potential trans-splicing acceptor sites. This could give rise to two isoforms of protein although the expression of the smaller variant seems to be dominant [16].

**Figure 3:**
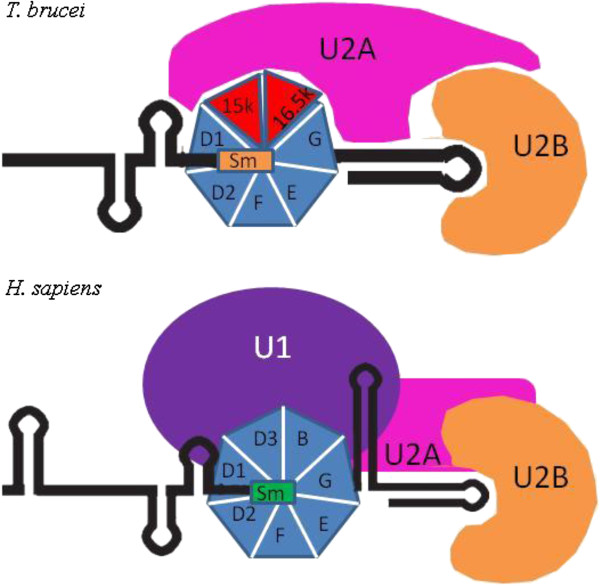
**The spliceosome of Trypanosomes compared to that of *H. sapiens.*** Unlike the spliceosome of most eukaryotes the SM protein complex of Trypanosomes contains two unique Sm proteins SM15K and SM16.5K. The Sm core associates with U2 and not U1 in Trypanosomes. The human stem loop RNA separating U2A and U1 is absent in Trypanosomes. The unique SM proteins are required for the correct assembly of U2A in Trypanosomes*.* The unique structure of the Trypanosome spliceosome seems to play a role in Trans-splicing, which is the dominant form of mRNA splicing in Trypanosomes*.*

The spliceosome of trypanosomes differs from that of most eukaryotes due to a less stringent conserved motif on the snRNA’s SM site. Two Sm proteins (SmB and SmD3) are replaced by two proteins that are specific to U2 (SM15K and SM16.5K) (Figure 3). The unique Sm proteins seem to be an absolute requirement for the correct assembly of U2 SnRNP, associating with U2 and not U1 [13,17]. They may also be important for the correct positioning of mRNA as trypanosomes do not make use of sequence complementarity to define branch point sequences [18]. The differences between the structure of the human and trypanosome spliceosome are not limited to these Sm proteins, as the human stem loop RNA separating U2A and U1 is absent in trypanosomes [17]. Genes in trypanosomes are also arranged in long polycistronic transcription units. These genes are then transcribed by RNA polymerase II beginning at switch regions and proceeding bidirectionally. The resulting RNA undergoes a specialised form of splicing known as trans-splicing with a different mRNA transcript [13].

#### Spliced leader trans-splicing

**Figure 4:**
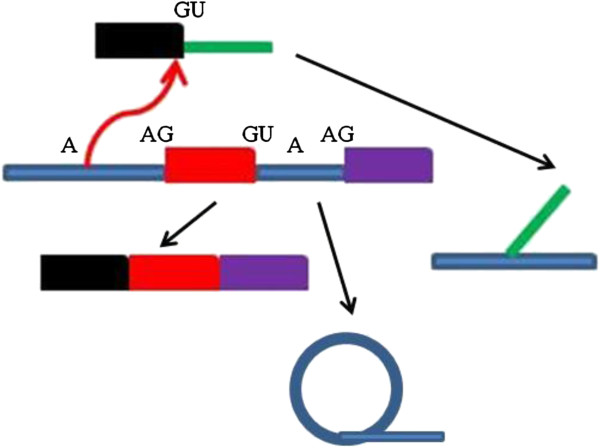
**Spliced Leader RNA trans-splicing.** The SL RNA (black) attaches to the first exon (red) of an mRNA. The outron of the mRNA and the intron of the SL RNA attach and form a branch in the same way that in traditional splicing the intron forms a lariat.

Another form of splicing known as trans-splicing, is used to generate a Short Leader sequence of RNA (the SL RNA), which performs the function of the U1 snRNP (Figure 4) [13]. As previously stated the Sm core associates with U2 and not U1 in trypanosomes and knockdown studies showed that U1 is not required for trans splicing activities [17,19]. With trans splicing being the most common form of splicing in trypanosomes, as most trypanosomal genes do not contain introns, with only two known examples in *T. brucei*[17]*.* Trypanosomes are able to successfully evade the host’s immune response by switching the expression of alleles coding for the Variant Surface Glycoprotein (VSG) [20]. In bloodstream forms of *T. brucei* 7-11% of all spliced transcripts code for VSG [15]. Variations in the surface antigen VSG are created through spliced leader trans-splicing [21]. This specific type of mRNA splicing leads to the replacement of the 5’ end of an mRNA transcript with a small nuclear mRNA referred to as an SL-RNA. The 3’ end of the trans-RNA contains a binding site for the Sm protein complex. SL trans splicing can provide a 5’ cap for protein coding RNAs when the RNA is transcribed by RNA polymerase 1 (Figure 4) [21]. It is also required to turn polycistronic RNA transcripts into individual monocistronic mRNA [22]. However, in terms of increasing the hyper variability of surface proteins of the parasite the most important role that can be played by SL trans-splicing is that of “trimming” or “sanitizing” the 5’ untranslated end of mRNA. By removing the 5’ end of the transcript it does not matter what sequence is present, and this would allow for less selective pressure on the 5’ end allowing for the evolution of the gene, by including new transcription start sites or to incorporate a wider variety of useful sequence components [22].

The ability to vary the VSG structure and avoid recognition by the host immune system depends on the trans-splicing of mRNAs encoded by genes that have been rearranged and duplicated. There are over 1000 VSGs encoded in the genome of Trypanosomes [23]. This form of alternative splicing will have important consequences for the cellular location of the peptide as it changes the N terminal targeting sequence. It may also play a role in changing the stability and half-life of the protein.

### Plasmodium

**Figure 5:**
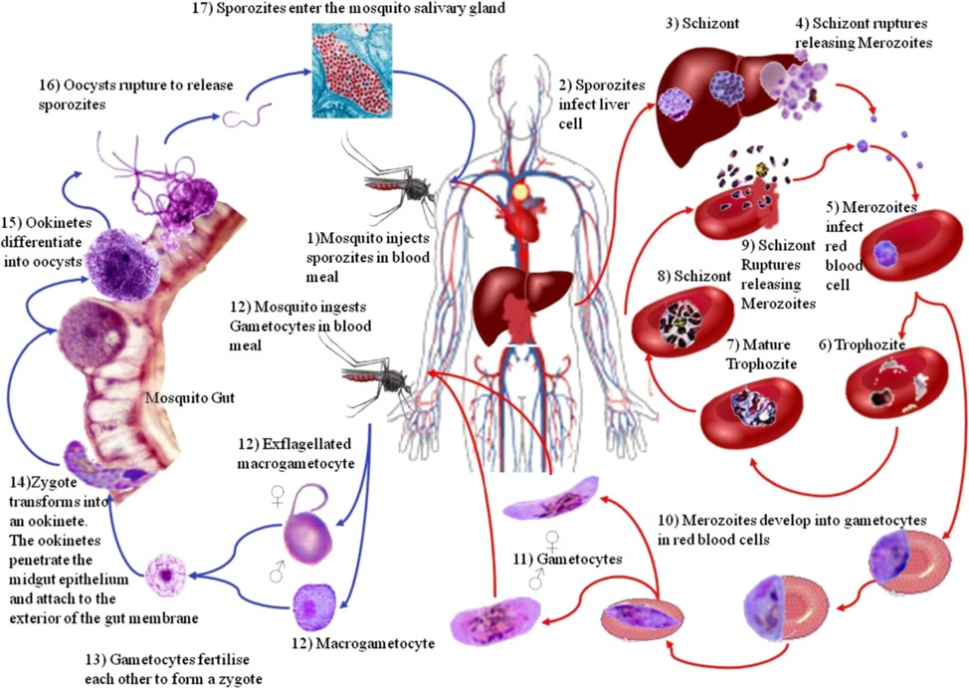
**Life cycle of *Plasmodium falciparum.*** The human host harbouring sexually immature parasite is marked using red arrows. Here the parasite multiplies asexually in the liver (exoerythrocytic schizogony) and in red blood cells (erythrocytic schizogony). The sporozoites that invade the liver become trophozoites, divide and develop into a liver schizont. These contain thousands of nuclei. The resulting merozoites infect blood cells and turn into trophozoites. These develop into schizonts with 8–32 nuclei which are released when the erythrocytes burst and can re-infect new erythrocytes. Erythrocytes that contain other stages stick to the endothelial cells of the blood capillaries, escaping destruction in the spleen, allowing gametocytes to be produced. The cycle in the mosquito which harbours sexually mature parasites is shown in blue. Here the sexually differentiated gametocytes change into gametes in the mosquito's gut, where fertilisation of the macrogamete occurs. Large oocysts, containing up to 10,000 sporozoites, develop on the outer surface of the gut wall. When they burst the sporozoites enter the mosquito's hemolymph, and ultimately reach the salivary glands.

For thousands of years malaria has persistently claimed a large number of human lives. In 1880 a French army physician, Charles Louis Alphonse Laveran, identified the causative agent of malaria, in the form of gametocytes of the protozoan parasite *Plasmodium falciparum.* The genus *Plasmodium* is made up of unicellular parasites that infect a wide range of hosts. These parasites replicate asexually and target the host’s erythrocytes leading to a stiffening of the erythrocyte membrane. *Plasmodium falciparum*, as the leading cause of malaria infection (75% of malaria), is considered the most dangerous parasite. One of the features of malaria is the inability of the host’s immune system to prevent infection with the re-infection of hosts occurring even in endemic malaria areas. Malaria parasites are able to evade the immune response of the host, firstly by going through most of its lifecycle (Figure 5) without presenting any antigen on the surface of infected cells and secondly by increasing the hyper-variability of the surface proteins. This is achieved via the amplification of extensive repertoires of multicopy hyper-variable gene families that encode infected erythrocyte or merozoite surface proteins [24]. The genome of *Plasmodium falciparum* consists of roughly 5700 genes and at 19% has the lowest GC content of any genome sequenced thus far [25]. Sequencing of RNA transcripts or cDNA libraries pointed to the fact that the transcriptome contained variable length un-translated regions as well as prevalent alternate splicing of transcripts [26]. In addition to this over 50% of the genes in *P. falciparum* contain introns [27]. Using current sequencing technologies four types of alternate splicing strategies were identified in *Plasmodium*. These were exon skipping, intron retention or creation and 3’ or 5’ alternate splicing arising from coordinate changes to exons or alternate start or stop codons [26]. Splicing of genes in *P. falciparum* was found to have effects on the localisation of the protein within the cell as splicing results in the removal of a signaling peptide. Similarly the cell localization can be changed by affecting the solubility of the protein via the removal of a trans-membrane domain. Splicing has also been observed to drastically alter protein function due to the removal of an active domain. Premature stop codon can also contribute to protein regulation by resulting in the abolishment in the expression of the mature protein [28].

#### Splicing in *plasmodium*

A putative homolog of the SF2/ASF SR splicing protein was identified in *Plasmodium*. This component of the spliceosome affects alternative splice site selection by antagonizing other SR proteins as well as functioning to stimulate transcription by binding to ribosomes [28]. In addition to this, a functional SR protein PfSR1 as well as the SR-protein specific kinase that regulates it (PfSRPK1) have also been identified [29]. The splicing branch points, that give rise to the excised lariat structure of the intron, are also unusual in *P. falciparum.* The 5’ splice site is poorly conserved and is able to still function with various substitutions. Some introns also have numerous branch points indicating a further mechanism for alternate splicing [30].

Alignment of a cDNA library from *P. falciparum* revealed that there were 363 splicing events that demonstrated splicing of intron-exon junctions from the antisense strand. The majority of these antisense transcripts mapped to genes that contain introns, implying that they may play a role in intron splicing [31]. Antisense splicing may play a role in the control and regulation of transcription of sense RNA. This can be accomplished through steric hindrance as two polymerases cannot function on opposite DNA strands. An antisense RNA molecule may bind to and hide a splicing site on the sense mRNA leading to alternate splicing [31].

#### Surface antigens

**Figure 6:**
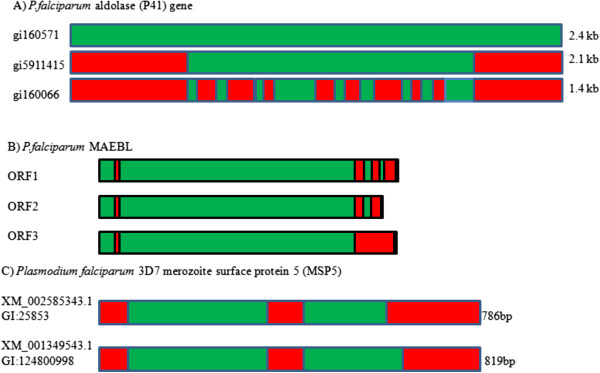
**Transcripts of alternatively spliced immune related genes in *Plasmodium. *(A)** The blood antigen protein P41-3 is alternatively spliced to give rise to three different transcripts of 2.4 kb, 2.1 kb, and 1.4 kb. **(B)** The MAEBL trans-membrane receptor binds erythrocytes and is expressed as different isoforms depending on the developmental stage of the parasite. The *maebl* gene consists of two open reading frames ORF1 and ORF2. Some of the isoforms are soluble unlike the typical trans-membrane MAEBL and these soluble isoforms are the result of an ORF2 transcript. **(C)** Merozoite surface proteins found on the surface of the merozoite *P. falciparum* contain three MSPs: MSP2, MSP4 and MSP5. The 40 kDa MSP5 is alternatively spliced using an alternate stop codon.

Various antigenic proteins produced by *Plasmodium* species are known to have isoforms that are the result of alternate splicing. The blood antigen protein P41-3 of *P. falciparum* is a 375 amino acid protein encoded by a 2137 bp gene. It was the first parasite gene where alternative splicing was observed (Figure 6) [32]. Alternative splicing of the mRNA gives rise to three different transcripts of 2.4 kb, 2.1 kb, and 1.4 kb. The exact role of this protein is not known, however it appears to be a soluble protein that is exported from the parasite [32]. The *P. falciparum* surface antigen UB05, which is recognized by IgG antibodies from adults that are semi-immune to malaria infection [33], was found to be alternatively spliced through 3’ and 5’ alternate splicing [26,34]. Another suspected surface antigen, PF 70, is soluble in Triton X, suggesting that it is membrane bound. The protein is a novel 70 kDa protein that reacts with anti *P. falciparum* and anti *P. yoelli* serum [35]. The mRNA coding for PF 70 contains a 5’ alternative splice site, resulting in a protein that is 25 amino acids shorter than the protein translated from the full length transcript [34].

Another class of surface antigens, Merozoite Surface Proteins (MSP) are proteins found on the surface of protozoan daughter cells. Interest has been expressed in the use of these proteins to create a malaria vaccine, as initial reports suggested that this protein is exposed on the surface of the merozoite and displays limited genetic diversity [36]. In *P. falciparum* chromosome 2 contains three *MSPs: MSP2, MSP4* and *MSP5*. MSP5 is a 40 kDa glycosylphosphatidylinositol (GPI)-anchored protein with an epidermal growth factor domain [37]. However, recently it was discovered that MSP5 is alternatively spliced using an alternate stop codon (Figure 6) [26]. This may then result in increased variation despite a high sequence similarity, thus allowing the merozoite to avoid the immune system of the host and decreasing its usefulness as a vaccine target.

Malaria is associated with the adherence of infected erythrocytes to the endothelial lining of blood vessels [38]. The Merozoite Apical Erythrocyte-Binding Ligand (MAEBL) trans-membrane receptor was identified in the blood stage of *P. falciparum.* The protein binds erythrocytes and unlike many proteins from *P. falciparum,* which seem to be stage specific, MAEBL is expressed in both sporozoites and merozoites. However, the *maebl* gene consists of two open reading frames ORF1 and ORF2. This results in *maebl* being expressed as different isoforms depending on the developmental stage of the parasite (Figure 6) [39]. Some of the isoforms are soluble unlike the typical trans-membrane MAEBL and these soluble isoforms are the result of an ORF2 transcript. Transcripts of this soluble MAEBL are only found in the oocyst stage. It is unknown what the significance of these alternatively spliced isoforms are [39]. Another protein involved in erythrocyte binding is Cytoadherence linked asexual gene 9 (clag9). This is a trans-membrane protein found on the surface of infected erythrocytes that is required to bind to the endothelial cell receptors [40]. By measuring antibody response to synthetic clag9, it was determined that this protein is immunogenic [41]. Clag9 was found to be alternatively spliced through 3’ and 5’ alternative splicing [26]. The exact roles played by the isoforms of these various protein antigens remains unknown.

In addition to these Otto [26] concluded that there were an additional 32 conserved unidentified proteins in *P. falciparum* that could be expressed as different isoforms due to alternate splicing. Another member of the phylum Apicomplexa that is closely related to *Plasmodium* is *Theileria annulata. Theileria* are able to reversibly transform their host’s cells. These parasites are tick borne and cause lympho-proliferative diseases of cattle – tropical theileriosis and East Coast fever (ECF) [42]. The gene coding for an immunogenic protein, that was used to establish a diagnostic ELISA assay, was given the name clone 5. This gene is alternately spliced at its single intron. This splicing is a combination of intron retention and 3’ splice site alteration due to the introduction of another stop codon. Both mRNAs are translated into a corresponding long and short protein [43].

#### Hyper-variable gene families

*Plasmodium* species express four families of hyper-variable genes. These genes have many copies in the genome. The first of these families is used to prevent the parasite being sent through the spleen and destroyed. The *P. falciparum* erythrocyte membrane protein 1 (PfEMP1) family, are large proteins ranging between 250 and 300 kDa. They function as cytoadhesion proteins which results in infected erythrocytes sticking to the walls of blood vessels and not cycling through the spleen. These proteins are encoded by a multicopy gene family named *var* which consists of about 60 genes, of these only a single protein is expressed at a time leading to a wide variety of antigenic phenotypes and cyto-adherent abilities [24]. The *Plasmodium vivax* homologs of the *var* family, named the *vir* family, display varying numbers of exons as well as alternate splicing [44]. *Var* gene expression seems to be regulated through chromatin remodeling as *var* expression is silenced by histone deacetylases and enhanced by histone acetylation [45]. Two separate promoters control *var* gene expression. An upstream promoter creates coding mRNA, and an internal intron promoter which creates a non-coding RNA. The intron promoter seems to be bi-directional. This promoter gives rise to short antisense mRNA that appears to associate with the *var* genes on the chromosome. In this way the antisense RNA may regulate the transcription of *var* gene products [45].

The other three families all contain a trafficking motif known as pexel/TS, which allows the protein to be transported to the cytoplasm of the infected erythrocyte. They also share the same gene architecture consisting of a short first exon encoding a signal peptide followed by a longer exon. All three families code for trans-membrane proteins of varying size but similar structure. They consist of a two trans-membrane domains joined by a variable loop. This loop contains the hypervariable region which is exposed on the outside of the erythrocyte to antibody mediated immune selection [24]. The *Pfmc-2TM* family consists of 13 genes; the subtelomeric variable open reading frame family (*stevor*) consists of 30–40 genes [24] and the repetitive interspersed family (*rif*) consists of over 150 copies. This makes the Rif family the largest gene family in *P. falciparum*[46]. There is evidence that both the *rif* and *stevor* genes are alternatively spliced.

Stevor is suspected to play a role in antigenic variation [47,48]. It is expressed in the late stages of the asexual forms of the parasite, where it gathers in structures known as the Maurer’s cleft. This structure plays a role in the release of parasites from erythrocytes [48]. The expression of the protein at only the late stages seems to imply that it plays a role in the release of the parasite from erythrocytes or alternatively it acts to shield other cleft components from the immune system of the host [48]. Stevor is also expressed in the gametocytes and it is here where alternate splicing gives rise to a truncated version that lacks one of the trans-membrane regions [48], implying changes in the proteins function of localisation. These *Stevor* transcripts have a diverse structure resulting from the use of alternate splice sites giving rise to frame shifts and deletions [49]. Mature stage gametocyte infected erythrocytes need to re-enter circulation in order to infect blood feeding mosquitoes. This is achieved by increasing the severity of membrane deformation. Stevor has been shown to play a role in erythrocyte membrane deformation [50] where an increase in the rate of disassociation of Stevor from the membrane surface occurs prior to increased membrane deformation [51]. A switch between the transcription of various isoforms, especially those with fewer trans-membrane regions, may aid in this dissociation. The Rif family was found to be 3’ and 5’ alternately spliced [26]. The ORF of the genes are also spliced into short signaling peptides [46].

#### Alternative splicing of genes in the immune response of the mosquito host

The salivary gland of the female mosquito is the primary site of infection by *Plasmodium* parasites. An analysis of the transcriptome of the salivary gland before and after infection revealed changes in the levels and identities of the transcripts. In the case of the D7 odorant binding protein (OBP) there is a change in the identity of the transcripts due to alternate splicing [52]. There are instances in which odorant receptor molecules have been identified as being involved in innate immune signalling systems [53]. Additionally expression of OBPs may be in response to oxidative stress where they act as scavengers of reactive oxygen species [54] and this may be important to protect the mosquito from the negative effects of its own immune response to the parasite. Infection with dengue virus increased the expression of OBP in *Aedes aegypt*i. This was shown to be a strategy by the virus to manipulate the behavior of the mosquito with an increase in the expression of OBPs leading to an increase in the frequency of the mosquito feeding, thereby increasing host infection [55].

**Figure 7:**
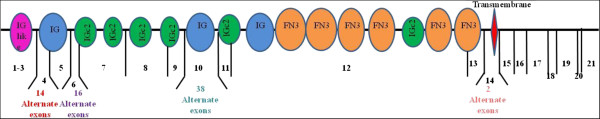
**Alternative splicing of the *Anopheles gambiae *Down syndrome cell adhesion molecule (DSCAM).** This Ig domain protein has multiple alternative splice exons within its IG regions. The above image shows the different domains found within the protein and the exons encoding these domains. Exons 4, 6, 10 and 14 are alternately spliced with 14, 16, 38 and 2 alternate exons respectively. The different combinations of these exons can give rise to 31 920 splice forms. The protein plays a pathogen recognition role, with the large number of isoforms allowing it to recognize, bind and respond to a wide array of different pathogens.

Molecules with a more direct function in the immune system whose transcription is altered following infection with malaria parasites include serpins [56], peptidoglycan recognition proteins [57] and Down syndrome cell adhesion molecule (Figure 7) [58]. Serpins play an important role in the regulation of the immune response. The classic example is the role played by the serpin Nec in down-regulating the Toll pathway by inhibiting the serine protease Persephone [59]. As a result of alternate splicing the Serpin10 gene of *Anopheles gambiae* can give rise to four splice variants due to alternate splicing. The transcription of two of these isoforms is increased following infection with *Plasmodium bergii* while bacterial infection had a different effect on the levels of each isoforms mRNA [56]. Serpin10 is also known as plasmodium-related inhibitory serine protease inhibitor [60]. Alternative splicing also gives rise to three isoforms of Serpin4 from *A. gambiae*[61]. The functional role of these isoforms is unknown. However, the *A. gambiae* Serpin10 and Serpin4 show the highest sequence similarity with the *Drosophila melanogaster* Serpin42Da and Serpin28Dc respectively. Serpin28Dc is suspected to play a negative role in the regulation of melanization, the defense response and to be involved in the wound response [62].

An *A. gambiae* a homolog of the Peptidoglycan Recognition Protein long form C was found to encode for three different isoforms which carry alternative PGRP domains linked to a common backbone consisting of a trans-membrane region and signaling peptide [57]. In *Drosophila* this protein is required for the detection of bacterial pathogens and initiates an immune response via the Imd pathway [63]. The mosquito isoforms are, however, differentially transcribed depending on the nature of the infectious agent [57]. The mosquito PGRP-LC detects bacterial infection but also detects *Plasmodium* and this detection results in an anti-parasitic defense response. The isoform responsible for this detection and subsequent response is PGRP-LC3 [64].

The Down syndrome cell adhesion molecule (DSCAM) is an Ig domain containing protein that was identified in *Drosophila* as playing a role in neuronal guidance. This molecule contains multiple alternative splice exons within its IG regions (84) that can give rise to 31 920 splice forms (Figure 7) [58,65]. It was first suspected to be involved in the immune response of insects when it was found to be expressed in immune related cells within *Drosophila* and was later identified to be an essential recognition molecule in the insects immune response [65]. The hyper variability of the molecule allows it to recognize, bind and respond to a wide array of different pathogens with differently spliced forms being expressed in response to different pathogens [58,65]. *Plasmodium* infection results in the increase in variability of exon 4 and 6 in particular. The increase in the variability of exon 6 also seems indicative of infection by multiple strains of *Plasmodium* with different genotypes [58]. In this way the mosquito can respond to the hyper variability of the pathogen.

### Schistosoma

**Figure 8:**
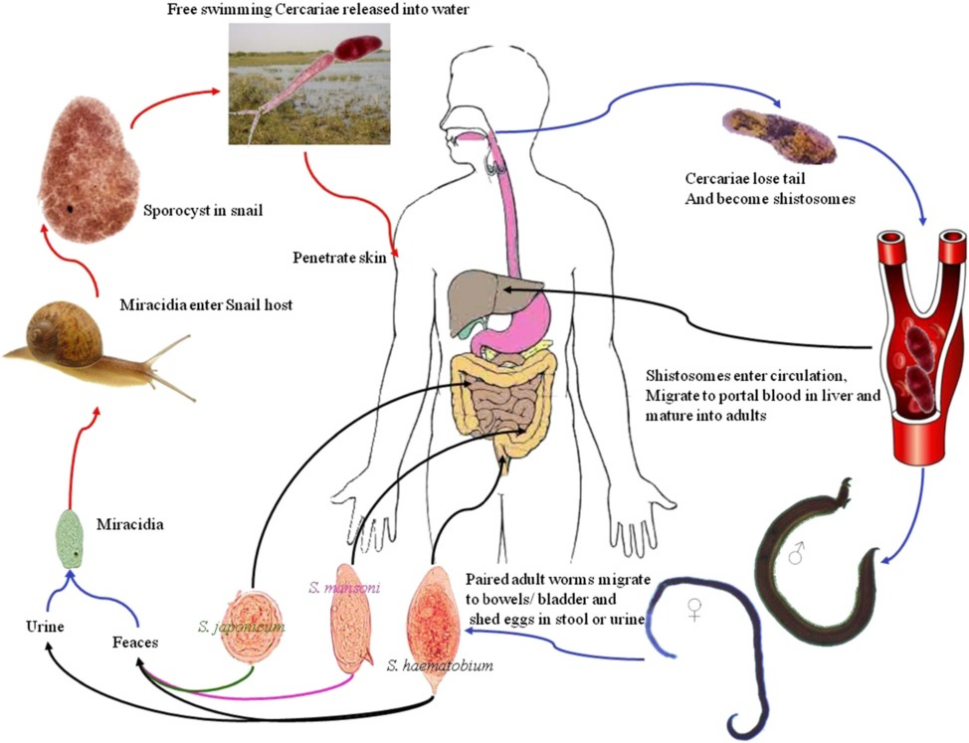
**Life cycle of Schistosomes*.*** Unlike other Trematodes, schistosome adults have two sexes and are located in blood vessels. Eggs from human hosts hatch into free swimming miracidia which infect specific snail species. These develop into two generations of sporocysts and result in the production of cercariae which are released from the snail and swim freely in water where they encounter and penetrate the skin of the human host. The cercariae shed their tails to become schistosomules, which enter small veins and develop into adult worms in mesenteric veins. Their exact location varies with species. *S. hematobium* occurs in the bladder venous plexus and rectal veins. Eggs are deposited in the small veins of the portal and perivesical systems. In *S. mansoni* (pink arrows) and *S. japonicum* (green arrows) the eggs migrate to the lumen of the intestine and are excreted in the feces. In *S. hematobium* (black arrows) eggs migrate towards the lumen of the intestine or the bladder and uterus and are eliminated with feces or urine.

The tropical disease schistosomiasis is caused by blood flukes of the genus *Schistosoma.* This disease affects 210 million people and is still a major source of morbidity, causing approximately 280, 000 deaths per year in sub-Saharan Africa. These worms require a vertebrate host and an intermediate gastropod host (Figure 8). Schistosomes are not hermaphroditic with male worms having a gynecophoral canal in which the female worm resides. The sequencing of the genome of *Schistosoma mansoni* revealed 11, 809 putative genes encoding 13,197 transcripts [66].

**Figure 9:**
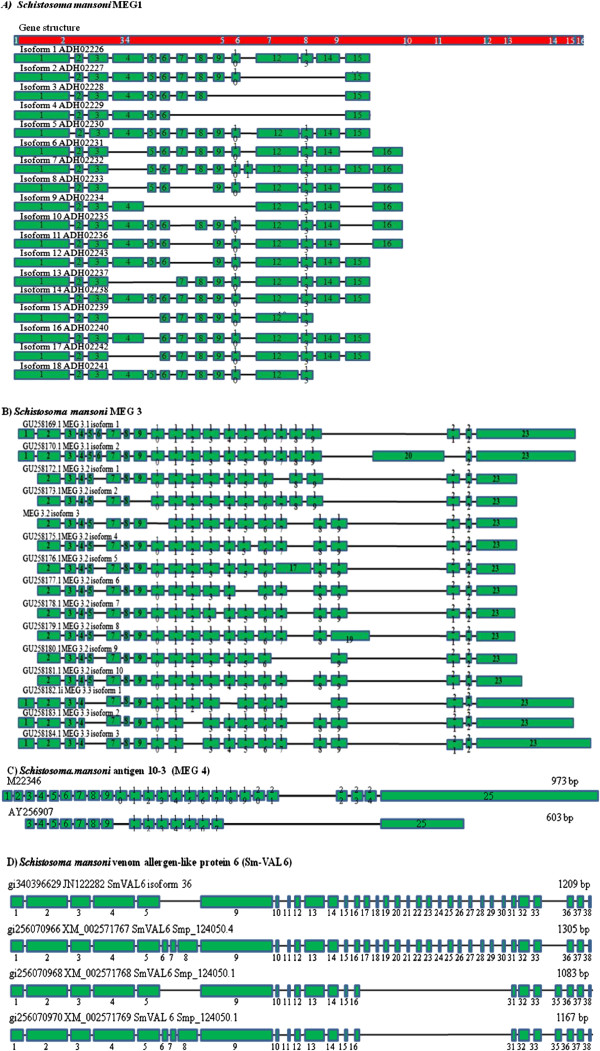
**Transcripts of alternatively spliced immune related genes in *Schistosoma mansoni*. (A)** MEG 1 has 18 isoforms. These isoforms display very lifestage specific transcription. The exact role of MEG1 is unknown. **(B)** MEG 3 has four members with three, 10, three, and two isoforms four family members with 3, 1 and 4 isoforms each. MEG 3 is also known as GRAIL. **(C)** The 10–3 surface antigen also known as MEG 4 has different transcription start sites that give rise to two isoforms that are expressed at different stages in the parasites lifecycle. **(D)** SmVAL6 contains 38 exons, the four largest code for conserved protein motifs, while the smaller exons are alternately spliced to give rise to at least 5 isoforms. SmVAL6 probably arose due to a recombination event between a MEG gene and a SmVAL gene.

The first report of alternate splicing in *S. mansoni* concerned the use of sera from humans infected with *S. mansoni* to recognise an antigen from esophageal gland of *S. mansoni.* This antigen was given the name 10–3 and is made up of multiple alternatively spliced 27 bp exons and 81 bp repeats [67]. Alternative splicing also occurs due to different transcription start sites and changes in mRNA processing. This alternative splicing gives rise to isoforms that are differentially expressed in different life stages of the parasite (Figure 9). The 10–3 gene contains tandem repeats and all transcripts share the same 3’ sequence. However, the sequence 5’ of the tandem repeats is variable [68].

At least 45 genes were identified that contained a gene structure consisting of multiple very small exons (micro-exon structure) (Figure 9). Although micro-exon genes (MEG) have been identified in other organisms, *S. mansoni* is unusual in that the micro exons can make up to 75% of the coding sequence of the gene. Most of these genes contain a signal peptide at the 5’ end, while three have membrane anchors. Sequencing of transcripts of MEGs from families *MEG-1, MEG-2* and *MEG-3* showed that there were up to seven splice variants that arose due to exon skipping [66,67]. The 10–3 antigen described above was identified as a MEG and given the alternate name of MEG-4 [67]. The initial number of micro exon containing genes (45) that were identified in *S. mansoni*[66] was upon further analysis expanded by another 27 genes giving a total of 72 genes consisting of microexons. These genes can be divided into 18 families, which can be further divided into two groups, based on the number of members they contain [67]. Further transcriptome sequencing may have identified a further 11 micro-exon containing genes [69]. Protein expression studies show these genes code for small proteins with isoforms that have very similar size and charge that appear to be secreted. These proteins are expressed in epithelial and gland tissue. 16 MEG protein products have also been detected in secretions or detected in the glands of *S. mansoni.* The transcription of these genes increases during the mammalian stages of the life cycle where the parasite may interact with the host’s immune system, and appear to be associated with host invasion and persistence within the host [67,70]. Another feature of MEGs which allows for the neat segmentation of the coding region is the fact that the exons are symmetrical. This allows for the excision and without altering the reading frame [67].

Despite the fact that some of these proteins may be membrane bound and therefore exposed to the external environment of the host; it is unknown whether they are antigens. However, as they are detected in secretions and seem to be directly associated with a particular life stage, it is likely that these proteins are being directly exposed to the host and its immune system [67]. Therefore, the ability to vary their structure may provide protection against immune recognition by the host through an antigenic variation based immune evasion strategy similar to that seen in trypanosomes and *Plasmodium*. However unlike the antigenic variation genes within those parasites, these MEGs are expressed simultaneously and not sequentially [67]. A gender based alternative splicing of a transcription co-factor that interacts with the spliceosome was identified in *S. mansoni.* The gene SmCA150 codes for a protein that influences the alternative splicing of other gene transcripts. This may form the basis for other differences observed in RNA splicing observed between males and females [71].

The *S. mansoni* venom allergen like (SmVAL) protein family was given this name due to the similarity of the proteins with wasp venom allergen [72]. All these proteins contain a sperm coating protein domain. This domain is found in proteins that play a role in the immune response, testes development, as a venom toxin and host invasion, where parasitic nematode species secrete SCP/TAPS proteins into the host during infection [72]. In plants, proteins containing this domain are involved in the immune response, while in mammals they are known to be involved in lung development [73].

29 SmVAL genes have been identified and can be divided into two groups. The first group codes for proteins that contain a signal peptide and appear to be excreted. The second group codes for proteins that do not contain a signal peptide and should play an intracellular role [72]. Group 1 VALs such as 1, 2, 3, 4, 5, 7, 8, 9, 10, 12, 14, 15, 18, 19, 20, 21, 22, 23, 24, 25, 26, 27 and 28) are associated with host invasion. SmVALs4 10 and 18 are associated with the invasion of the mammalian host. The other group 1 VALs appear to be involved with the invasion of the molluscan host. The role of most group two SmVALs such 6, 11, 13, 16 and 17 are unknown. However SmVAL6 is a group 2 VAL expressed during the mammalian life stages [73]. The presence of VALs in parasitic and non-parasitic helminthes indicates that they may play a role in life cycle changes [73].

Through the use of Rapid Amplification of cDNA Ends (RACE) mRNA isoforms were identified for multiple SmVALs. These included *SmVAL1, SmVAL2, SmVAL4, SmVAL6, SmVAL7* and *SmVAL11*[72]. *SmVAL6* was shown to contain micro-exons with alternative use of these exons giving rise to the different isoforms [72]. These isoforms were expressed at different lifestages of the parasite. For instance *SmVAL1, SmVAL2* and *SmVAL4* isoforms were isolated from mixed sex cercariae RNA while *SmVAL6, SmVAL7* and *SmVAL11* isoforms were isolated from mixed sex, 7-week adult RNA [72]. One of the members of this family *SmVAL6* contains four exons of an average size and code for the conserved motifs in the protein family (Figure 9). However, this is followed by seven small exons, which is similar to the structure of MEGs. As many as 5 isoforms have been identified that occur due to alternate splicing of these smaller exons. SmVAL6 probably arose due to a recombination event between a MEG gene and a SmVAL gene. Additionally, both proteins are excreted and therefore will probably interact with the host’s immune system and generate an immune response [72]. The antigenic role played by different SmVALs was found to vary amongst individual family members. For example despite SmVAL4 and SmVAL26 both being secreted group 1 VALs, SmVal4 elicits an immune response in mice while smVAL26 does not, as assessed by a mouse model of airway inflammation. This difference in members of this family to elicit an immune response is most likely based on the localisation of the protein within the parasite. SmVAL4 and SmVAL26 are both group 1 SmVALs and are secreted. Transcription and expression data show that SmVAL4 is excreted during mammalian host skin invasion and is released during the cercariae stage, while SmVAL26 is excreted from the egg (Figure 8) [74].

The polymorphic mucins (SmPoMucs) are a protein family consisting of approximately ten members in *S mansoni.* The gene structure consists of between 0 and 20 tandem repeats of a 27 base pair exon near to the 5’ end of the genes. Alternative splicing as well as trans-splicing between transcripts generates hyper variability. Splicing also affects the proteins post-translational modification [75]. The mollusc host produces a highly variable immune receptor Fibrinogen Related Protein 3 (FREP3) that is required for host resistance to infection by *S. mansoni*[76,77]*.* These FREP3s interact and form complexes with the SmPoMucs [76]. Therefore the variation produced by alternate splicing plays a role in avoiding recognition by the invertebrate host’s immune system.

## Parasites, host immunity and HIV

Like the immune response against most infectious agents, the immune response to parasites includes non-specific factors, such as the genetic factors of the host, and specific immune responses. Cellular immunity appears to be the most important immune response against parasites such as *Plasmodium* while antibody based responses are the most important when dealing with trypanosomes. Cytokine production by the different subsets of T cells is important in the immune response and patient pathology [78]. Different subsets of helper and cytotoxic T cells are activated in response to different parasite infections. The Th1 cells respond through the production of interferon (IFN)-interleukin (IL)-12 and results in the activation of monocytes and macrophages and CD8+ cytotoxic cells. The Th2 cells produce IL-4, IL-5, IL-13 and results in antibody production [79].

**Figure 10:**
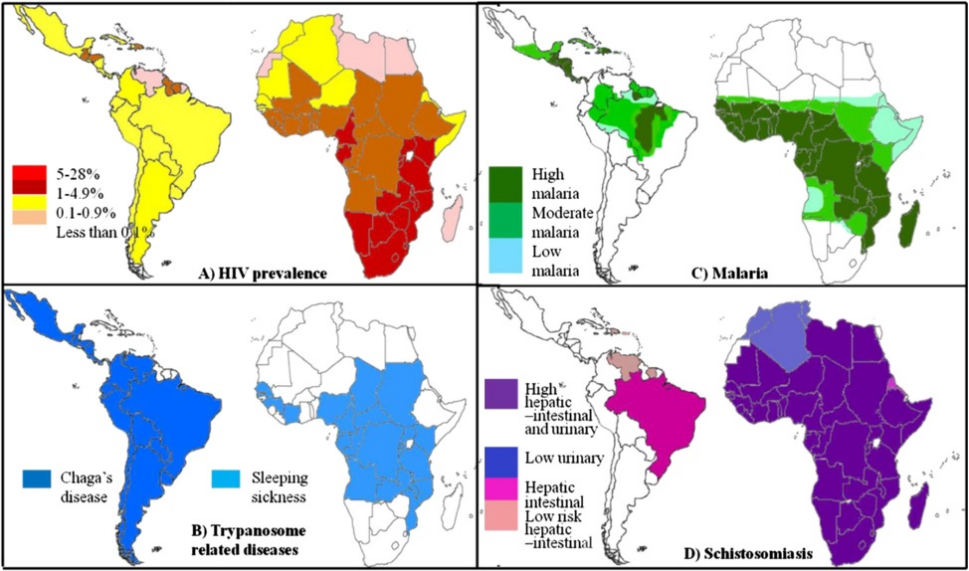
**The overlap in the geographical location of high HIV prevelance and parasitic diseases.** The above maps of South America and Africa show the prevelance of HIV infection **(A)** and the occurrence of the parasitic infections by *T. cruzi* and *T. brucei***(B)**; malaria causing *Plasmodium* species **(C)** and *Schistosoma* parasites **(D)**. These maps demonstrate that there is a geographical overlap amongst these infection. This implies that the increase in HIV prevelance observed in these areas may partly be due to the effect these paerasites have on host immunity.

The ideal situation for these eukaryotic parasites is to exist in the host at a level where infection persists without resulting in the death of the host. This chronic infection will result in a high incidence of immunopathology [78]. Patients with an immunocompromised immune system resulting from immunosuppressant drugs, chemotherapy or HIV infection show impaired CD4+ cell mediated immune response and an exacerbation of disease associated pathology [79]. Due to the overlap in the geographical locations of the highest incidence of HIV and most of the protozoan parasite infections (Figure 10), the combined effect of parasitic and HIV infections has proved to be an important consideration for diagnosis, treatment and prognosis of these infections.

**Figure 11:**
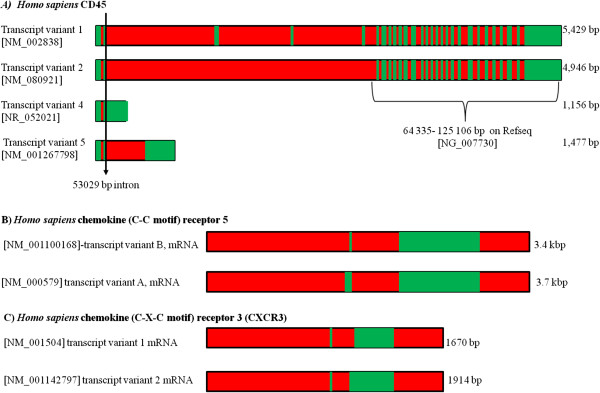
**Alternative splicing of cell surface receptors in *Homo sapiens *that play a role in HIV and parasite infections. (A)** Sequence data was available to construct a diagram showing splicing for 4 CD45 isoforms. These isoforms arise due to the alternate splicing of introns 4–6. The resulting mRNAs differ drastically in size and sequence. The *CD45* gene is 125 kbp in size with a large intron between exons 2 and 3. Isoforms 1 and 2 contain multiple exons dispersed across a large 60 kbp stretch of DNA. **(B)** Two CCR5 arise due to alternative splicing of a transcript derived from an alternative upstream promoter. The larger isoform negatively controls the expression of the smaller isoform. **(C)** Two CXCR3 isoforms have been identified which interact with the same chemokines but, this interaction results in opposing signaling based on the isoform present.

Infections with trypanosomes [80], malaria [81] and *Schistosoma*[82] are known to complicate the diagnosis of HIV infection using rapid diagnostic tests, due to the parasites inducing polyclonal B cell activation. This results in the production of a wide array of non-specific antibodies that can result in false positive diagnoses [80]. Chronic malaria infection also results in the increase in the levels of HIV RNA as well as a decrease in CD4+ cell number while HIV infection results in the increase in the severity of bouts of malaria [83]. *Schistosoma* infections accelerate HIV disease progression and transmission. This is largely due to physical damage in the urinary tract of the host as a result of the presence of the parasites eggs [12]. *Schistosoma* co-infection of Rhesus Macaques infected with Chronic Simian-Human Immunodeficiency Virus Clade C led to an increase in viral RNA expression and lower CD4^+^ counts [84]. *S. mansoni* infection leads to the increase in the number of cytokines cell surface receptors which can be used by HIV to infect a cell. The parasite also suppresses the Th1 immune response needed for the immune response to HIV [12,85]. For instance *Schistosoma* infection leads to increased CXCR4 expression levels on individual cells, but does not increase the number of cells that express this receptor. However, treatment of schistosomiasis leads to a decrease in the level of CCR5 and CXCR4 on CD4+ cells in both HIV infected and uninfected patients. Schistosomiasis was also found to lead to increased susceptibility to HIV infection, while treatment decreases the susceptibility to HIV infection and may slow disease progression [85]. Infection with *T. brucei* prior to HIV infection can however, decrease the viral infectivity, by inhibiting the ability of the virus from entering cells [86]. These interactions between parasites and HIV that alter infectivity and lowers the prognosis for AIDS appear to occur mainly at the level of the cell signaling molecules and cytokines. This may be due to the increase in the levels of cytokines such as (IFN)-γ, interleukin (IL)-12 and TNF, which can stimulate HIV replication [83], the inhibition of IL-2 synthesis [87] increase in CCR5 and CXCR4 synthesis [12]. Many of these cytokines, such as CD4, CD8 and CTLA4, are expressed as different isoforms, with the alternative splicing patterns for these genes changing in response to antigen levels (Figure 11) [88].

### CD45

At least 5 different CD45 isoforms are expressed due to the alternate splicing of introns 4–6. These isoforms range in size from about 180–240 kDa (Figure 11) with the levels of the lower molecular weight isoforms increasing after the T cell has been exposed to an antigen. These T cells with higher levels of the low molecular weight forms also have a higher turnover rate [89,90]. These CD45 isoforms may also differ in their ability to promote IL-2 secretion, their ability to associate with CD4 and CD8 and their ability to promote B cell maturation. Evidence does exist for the homodimerisation of CD45 which may also regulate CD45 function further through the homodimerisation of different isoforms [90]. It has also been noted that the expression of these isoforms may be dependent on the type of cell or the state of cell activation and differentiation [91], while HIV infection results in reduced surface expression of all CD45 isoforms [92].

The *T. evansi* parasite that causes surra disease in animals possesses a membrane bound toxic protein that kills lymphocytes in a CD45 dependent manner. The presence of CD45 isoforms may explain why only some of the population of peripheral blood mononuclear cell (PBMCs) died after they were treated with *T. evansi* protein extracts [91]. Like HIV, trypanosome parasites are further able to suppress the immune system of their mammalian hosts via an increase in the apoptotic killing of T cells [87]. Infection with trypanosome parasites induces a strong CD8^+^ T cell response in mammalian hosts. These cells predominantly recognise the trans-sialdase epitope [93]. *T. cruzi* and *T. brucei* do not synthesize their own sialic acid for inclusion into their surface receptors. Instead they make use of the enzyme Trans-Sialidase found on the surface of the parasites, to transfer sialic residues from the sialic-glycosylates of the host. This enzyme is also shed into the bloodstream where it leads to the induction of apoptosis in immune related cells [94]. This is a result of the sialylation of surface receptors such as CD4, CD8 and CD45 [94,95]. The different isoforms of CD45 are the main targets of Trans Sialidase, which may result in changes to the functions of the different isoforms due to changes in glycosylation [94].

Chronic *P. falciparum* exposure results in atypical memory B cells (MBC) that express increased levels of CD45RA as well as negative regulators of T cell response Programmed Death 1 (PD-1) and lymphocyte-activation gene-3 (LAG-3) [96]. Atypical MBCs are also a characteristic of HIV infection. These MBCs are less responsive to infection and are thus known as exhausted CD8^+^ T cells [97]. Naïve T cells are known to express higher CD45-RA [98] and this combined with the expression of the negative regulators of T-cell activation, suggests another means for *P. falciparum* to evade the immune response of the host.

### CCR5

The chemokine receptor CCR5 found on the surface of white blood cells plays a central role in HIV infection, where it acts as a co-receptor allowing the virus to enter target cells. Multiple isoforms of CCR5 arise due to alternative promoter regions as well as mRNA splicing (Figure 11). Of particular interest is two isoforms that arise due to the alternative splicing of a transcript derived from an upstream promoter [99]. Studies indicated that the longer isoform plays a role in negatively regulating the expression of the shorter isoform. Further isoforms that arise due to processing of transcripts from a downstream promoter may also play a role in expression by changing the stability of the mRNA [99]. HIV is too recent a human pathogen to have exerted any selective pressure on the various isoforms. However, pathogens that have infected humans for longer may have played an important role in exerting a selective pressure on the function and occurrence of these regulatory isoforms [100]. CCR5 isoforms that are unstable or down regulate the expression of active CCR5, result in an increased resistance to HIV and *Plasmodium vivax* infection [101]. CCR5 expression levels also influences the expression of IL-2 as well as the development of mature memory T-cells. These cells have higher levels of IL-2 and display an increased susceptibility to HIV infectivity [102]. Chronic *T. cruzi* infection, resulting in chronic Chagas disease can lead to cardiomyopathy due to the immune response damaging the tissues of the heart. This cardiomyopathy is accompanied by an increase in the expression of CCR5 on the surface of circulating lymphocytes and the use of a partial CCR5 antagonist protected mice from cardiomyopathy [103]. However, *T. brucei* infections can inhibit the entry of HIV into monocyte derived macrophages (MDM) partly due to a decrease in the expression of CCR5 [86]. Treatment of schistosomiasis leads to a decrease in the level of CCR5 and CXCR4 on CD4+ cells in both HIV infected and uninfected patients [85].

### CXCR3

Expression of the chemokine CXCR3 is increased following *Schistosoma* infection. CXCR3 is primarily expressed on NK cells and activated T lymphocytes. Two isoforms of CXCR3, generated by alternative splicing (Figure 11), allow many of the chemokines known to interact with CXCR3 (CXCL-9, CXCL10 and CXCL11) to play opposing roles when it comes to cell proliferation. The original isoform named CXCR3-A results in an increase in cell proliferation while CXCR3-B increases apoptosis levels. CXCR3 expression and function is up-regulated following *Schistosoma* infection [104] while mice lacking active CXCR3 showed an increased pathology associated with *Leishmania major* infection [105].

Cerebral malaria is a form of encephalopathy that results from the introduction of inflammatory molecules into the Central Nervous System via a damaged blood brain barrier (BBB). Damage to the BBB is caused by the adherence of parasitised red blood cells to the endothelial layer of the barrier [106]. Studies using CXCR3 knockout mice have demonstrated that this damage is mediated by CD8^+^ T cells which are recruited by CXCR3, and CXCR3^-^ mice fail to develop cerebral malaria [107,108]. The CXCR3 ligands CXCL9 and CXCL10 are also required for the development of cerebral malaria [108], but they are expressed in a tissue dependent manner with microglia having increased levels of CXCL9 and astrocytes having increased levels of CXCL10, while the levels of both were elevated in neurons [107,108]. A similar situation was observed following the intracranial infection of mice with *Toxoplasma gondii.* Here two populations of microglia formed in response to the infection, one containing the endotoxin response protein immunoresponsive gene 1 (IRG1) and CCR9; the other lacks IRG but expresses CXCR3. The IRG1^+^/CCR9^+^ microglia is neurotoxic and resistant to apoptosis while the IRG^-^/CXCR3^+^ microglia are neuro-supportive and sensitive to apoptosis [109]. The chronic activation of microglia in response to infection appears to be responsible for the neurodegeneration observed during AIDS. HIV targets microglia, which appear to be the only cells in the CNS which are productively infected with the virus, infected microglia then release neurotoxin mediators that result in the death of neurons and encephalopathy [110,111]. Low infection rates are observed in astrocytes and these may play the role of viral reservoirs [111]. The different roles played by CXCR3 in different infection and different stages of the infections may be governed by the various expression of one of the two isoforms. This would change the sensitivity of the cells to apoptotic stimuli and would allow the microglia to survive and act as a viral reservoir in HIV as well as allowing varied responses to different cytokines during the progression of cerebral malaria.

## Conclusion

Despite decades of research there is currently no vaccine to any human parasite. One of the major reasons why vaccines seem to fail during phase II trials is the diversity of the parasite. Not only does the hypervariability of surface proteins make it difficult to target, but parasites can also show the ability to rapidly develop drug resistance due to rapid evolution. Immune evasion of parasites through antigenic variation is a complex process with a vast amount of literature devoted to it; however, the vast majority of studies and reviews examine antigenic variation through the expression of different members of a multigene family and not through the alternate splicing of mRNA. The control of RNA transcription is poorly understood within *Trypanosoma* and *Plasmodium.* With the genomic sequences of these parasites and a better understanding of the role played by Trans SL RNA splicing and transcriptional control through antisense RNA, we are gaining a better picture of gene regulation in these parasites. It is important to understand the role played by the expression of different isoforms of antigens in immune evasion as these isoforms may offer new targets for therapy. This would involve the preferential selection of one isoform over another. This could be beneficial by leading to a either a decrease in immune evasion or a decrease in parasite viability. As many isoforms are expressed in a life stage specific manner, the expression of unique sets of genes at different life stages of the parasite points to a useful strategy involving the targeting of one of these particular stages, interfering in the progression from one life stage into another. Alternatively the life stage expression of different isoforms could be used as a diagnostic tool.

The geographical co-localisation of parasite infections by members of the genera *Plasmodium*, *Schistosoma* and *Trypanosoma* with a high prevalence of HIV infection, suggests that infection with parasites contributes to HIV infection and disease progression. HIV and parasite infection both lead to a suppression of the cellular immune system. These pathogens interact with the cellular immune system at the level of cell surface receptors and cytokines. The alternate splicing of the transcripts for these genes leads to drastic changes in their regulation and function. This may alter the way that parasites and HIV interact with the host’s immune system and with each other at a molecular level. Once again the preferential expression of some isoforms over others may have therapeutic effects in the simultaneous treatment of these infections.

## Abbreviations

clag9: Cytoadherence linked asexual gene 9; DSCAM: Down syndrome cell adhesionmolecule; FREP3: Fibrinogen related proteins; GPI: Glycosylphosphatidylinositol; LAG-3: lymphocyte-activation gene-3; MBC: Memory B cells; MSP: Merozoite surface proteins; MEG: Micro-exon gene; MDM: Monocyte derived macrophages; PfEMP1: *P falciparum* erythrocyte membrane protein1; PD-1: Programmed death 1; SL RNA: Short leader sequence of RNA; SmPoMucs: *S. mansoni* polymorphic mucins; SmVAL: *S. mansoni* venom allergen like; snRNAs: Small nuclear RNAs; stevor: Subtelomeric variable open reading frame family; VSG: Variant surface glycoprotein.

## Competing interests

The authors declare that they have no competing interests.

## Authors’ contributions

RH wrote the manuscript and ZD reviewed it critically. Both authors read and approved the final version of the manuscript.

## References

[B1] Marchetti G Pinotti M Lunghi B Casari C Bernardi F Functional genetics Thromb Res 2012 129 336 340 10.1016/j.thromres.2011.10.028 22100315

[B2] Black DL Mechanisms of alternative pre-messenger RNA splicing Annu Rev Biochem 2003 72 291 336 10.1146/annurev.biochem.72.121801.161720 12626338

[B3] Will CL Luhrmann R Spliceosome structure and function Cold Spring Harb Perspect Biol 2011 3 a003707 21441581 10.1101/cshperspect.a003707 PMC3119917

[B4] Bhopale GM Pathogenesis of toxoplasmosis Comp Immunol Microbiol Infect Dis 2003 26 213 222 10.1016/S0147-9571(02)00058-9 12676122

[B5] Restrepo BI Alvarez JI Castańo JA Arias LF Restrepo M Trujillo J Colegial CH Teale JM Brain granulomas in neurocysticercosis patients are associated with a Th1 and Th2 profile Infect Immun 2001 69 4554 4560 10.1128/IAI.69.7.4554-4560.2001 11401999 PMC98532

[B6] Hill DR Hewlett EL Pearson RD Lectin binding by *Giardia lamblia* Infect Immun 1981 34 733 738 6895883 10.1128/iai.34.3.733-738.1981 PMC350932

[B7] Peterson KM Alderete JF Acquisition of alpha 1-antitrypsin by a pathogenic strain of *trichomonas vaginalis* Infect Immun 1983 40 640 646 6601623 10.1128/iai.40.2.640-646.1983 PMC264902

[B8] Bloom BR Games parasites play: how parasites evade immune surveillance Nature 1979 279 21 26 10.1038/279021a0 88015

[B9] Schmid-Hempel P Immune defence, parasite evasion strategies and their relevance for ‘macroscopic phenomena’ such as virulence Philos T R Soc B 2009 364 85 98 10.1098/rstb.2008.0157 PMC2666695 18930879

[B10] Nissapatorn V Sawangjaroen N Parasitic infections in HIV infected individuals: diagnostic & therapeutic challenges Indian J Med Res 2011 134 878 897 10.4103/0971-5916.92633 22310820 PMC3284096

[B11] Andreani G Lodge R Richard D Tremblay MJ Mechanisms of interaction between protozoan parasites and HIV Curr Opin HIV AIDS 2012 7 276 282 22418447 10.1097/COH.0b013e32835211e9

[B12] Mbabazi PS Andan O Fitzgerald DW Chitsulo L Engels D Downs JA Examining the relationship between urogenital Schistosomiasis and HIV infection PLoS Neglect Trop D 2011 5 e1396 10.1371/journal.pntd.0001396 PMC3232194 22163056

[B13] PreuBer C Jae N Bindereif A mRNA splicing in trypanosomes Int J Med Microbiol 2012 302 221 224 10.1016/j.ijmm.2012.07.004 22964417

[B14] Berriman M Ghedin E Hertz-Fowler C Blandin G Renauld H Bartholomeu DC Lennard NJ Caler E Hamlin NE Haas B Böhme U Hannick L Aslett MA Shallom J Marcello L Hou L Wickstead B Alsmark UC Arrowsmith C Atkin RJ Barron AJ Bringaud F Brooks K Carrington M Cherevach I Chillingworth TJ Churcher C Clark LN Corton CH Cronin A The genome of the African trypanosome *Trypanosoma brucei* Science 2005 309 422 10.1126/science.1112642 16020726

[B15] Nilsson D Gunasekera K Mani J Osteras M Farinelli L Baerlocher L Roditi I Ochsenreiter T Spliced leader trapping reveals widespread alternative splicing patterns in the highly dynamic transcriptome of *Trypanosoma brucei* PLoS Pathog 2010 6 e1001037 10.1371/journal.ppat.1001037 20700444 PMC2916883

[B16] Penha LL Mendonça-Previato L Previato JO Scharfstein J Heise N Lima APCA Cloning and characterization of the phosphoglucomutase of *Trypanosoma cruzi* and functional complementation of a Saccharomyces cerevisiae PGM null mutant Glycobiology 2005 15 1359 1367 10.1093/glycob/cwj023 16037487

[B17] Günzl A The Pre-mRNA splicing machinery of trypanosomes: complex or simplified? Eukaryot Cell 2010 9 1159 1170 10.1128/EC.00113-10 20581293 PMC2918933

[B18] Lücke S Jürchott K Hung L-H Bindereif A MRNA splicing in Trypanosoma brucei: branch-point mapping reveals differences from the canonical U2 snRNA-mediated recognition Mol Biochem Parasit 2005 142 248 251 10.1016/j.molbiopara.2005.04.007 15923047

[B19] Tschudi C Ullu E Destruction of U2, U4, or U6 small nuclear RNA blocks trans splicing in trypanosome cells Cell 1990 61 459 466 10.1016/0092-8674(90)90527-L 1692263

[B20] Horn D McCulloch R Molecular mechanisms underlying the control of antigenic variation in African trypanosomes Curr Opin Microbiol 2011 13 700 705 20884281 10.1016/j.mib.2010.08.009 PMC3117991

[B21] Stover NA Kaye MS Cavalcanti ARO Spliced leader trans-splicing Curr Biol 2006 16 R8 R9 10.1016/j.cub.2005.12.019 16401417

[B22] Hastings KEM SL trans-splicing: easy come or easy go? Trends Genet 2005 21 240 247 15797620 10.1016/j.tig.2005.02.005

[B23] Robinson NP Burman N Melville SE Barry JD Predominance of duplicative VSG gene conversion in antigenic variation in African trypanosomes Mol Cell Biol 1999 19 5839 5846 10454531 10.1128/mcb.19.9.5839 PMC84433

[B24] Dzikowski R Templeton TJ Deitsch K Variant antigen gene expression in malaria Cell Microbiol 2006 8 1371 1381 10.1111/j.1462-5822.2006.00760.x 16848786

[B25] Aravind L Iyer LM Wellems TE Miller LH Plasmodium biology: genomic gleanings Cell 2003 115 771 785 10.1016/S0092-8674(03)01023-7 14697197

[B26] Otto TD Wilinski D Assefa S Keane TM Sarry LR Böhme U Lemieux J Barrell B Pain A Berriman M Newbold C Llinás M New insights into the blood-stage transcriptome of *Plasmodium falciparum* using RNA-Seq Mol Microbiol 2010 76 12 24 10.1111/j.1365-2958.2009.07026.x 20141604 PMC2859250

[B27] Gardner MJ Hall N Fung E White O Berriman M Hyman RW Carlton JM Pain A Nelson KE Bowman S Paulsen IT James K Eisen JA Rutherford K Salzberg SL Craig A Kyes S Chan MS Nene V Shallom SJ Suh B Peterson J Angiuoli S Pertea M Allen J Selengut J Haft D Mather MW Vaidya AB Martin DM Genome sequence of the human malaria parasite *Plasmodium falciparum* Nature 2002 419 498 511 10.1038/nature01097 12368864 PMC3836256

[B28] Iriko H Jin L Kaneko O Takeo S Han E-T Tachibana M Otsuki H Torii M Tsuboi T A small-scale systematic analysis of alternative splicing in *Plasmodium falciparum* Parasitol Int 2009 58 196 199 10.1016/j.parint.2009.02.002 19268714

[B29] Eshar S Allemand E Sebag A Glaser F Muchardt C Mandel-Gutfreund Y Karni R Dzikowski R A novel *Plasmodium falciparum* SR protein is an alternative splicing factor required for the parasite’s proliferation in human erythrocytes Nucleicl Acids Res 2012 40 9903 9916 10.1093/nar/gks735 PMC3479193 22885299

[B30] Zhang X Tolzmann CA Melcher M Haas BJ Gardner MJ Smith JD Feagin JE Branch point identification and sequence requirements for intron splicing in *Plasmodium falciparum* Eukaryot Cell 2011 10 1422 1428 10.1128/EC.05193-11 21926333 PMC3209046

[B31] Lopez-Barragan MJ Lemieux J Quinones M Williamson KC Molina-Cruz A Cui K Barillas-Mury C Zhao K Su XZ Directional gene expression and antisense transcripts in sexual and asexual stages of *Plasmodium falciparum* BMC Genomics 2012 12 587 22129310 10.1186/1471-2164-12-587 PMC3266614

[B32] Knapp B Nau U Hundt E Küpper HA Demonstration of alternative splicing of a pre-mRNA expressed in the blood stage form of *Plasmodium falciparum* J Biol Chem 1991 266 7148 7154 1707880

[B33] Kehdinga Titanji Vincent P Amambua-Ngwa A Anong Damian N Mbandi Stanley K Tangie E Tening I Yengo R Isolation and expression of UB05, a *Plasmodium falciparum* antigen recognised by antibodies from semi-immune adults in a high transmission endemic area of the Cameroonian rainforest Clin Chem Lab Med 2009 47 1147? 19728857 10.1515/CCLM.2009.255

[B34] Sorber K Dimon MT DeRisi JL RNA-Seq analysis of splicing in *Plasmodium falciparum* uncovers new splice junctions, alternative splicing and splicing of antisense transcripts Nucleic Acids Res 2011 39 3820 3835 10.1093/nar/gkq1223 21245033 PMC3089446

[B35] Ma HW Ray P Dhanda V Das PK Paliwal S Sahoo N Patra KP Das LK Singh B Kironde FAS A novel 70-kDa triton X-114-soluble antigen of *Plasmodium falciparum*That contains interspecies-conserved epitopes Exp Parasitol 1996 83 322 334 10.1006/expr.1996.0080 8823249

[B36] Goschnick MW Black CG Kedzierski L Holder AA Coppel RL Merozoite surface protein 4/5 provides protection against lethal challenge with a heterologous malaria parasite strain Infect Immun 2004 72 5840 5849 10.1128/IAI.72.10.5840-5849.2004 15385485 PMC517552

[B37] Black CG Wang L Hibbs AR Werner E Coppel RL Identification of the *Plasmodium chabaudi* homologue of merozoite surface proteins 4 and 5 of *Plasmodium falciparum* Infect Immun 1999 67 2075 2081 10225857 10.1128/iai.67.5.2075-2081.1999 PMC115940

[B38] Barnwell JW Asch AS Nachman RL Yamaya M Aikawa M Ingravallo P A human 88-kD membrane glycoprotein (CD36) functions *in vitro* as a receptor for a cytoadherence ligand on *Plasmodium falciparum*-infected erythrocytes J Clin Invest 1989 84 765 772 10.1172/JCI114234 2474574 PMC329717

[B39] Singh N Preiser P Rénia L Balu B Barnwell J Blair P Jarra W Voza T Landau I Adams JH Conservation and developmental control of alternative splicing in maebl among malaria parasites J Mol Biol 2004 343 589 599 10.1016/j.jmb.2004.08.047 15465047

[B40] Trenholme KR Gardiner DL Holt DC Thomas EA Cowman AF Kemp DJ clag9: A cytoadherence gene in *Plasmodium falciparum* essential for binding of parasitized erythrocytes to CD36 Proc Natl Acad Sci USA 2000 97 4029 4033 10.1073/pnas.040561197 10737759 PMC18136

[B41] Trenholme KR Boutlis CS Kuns R Lagog M Bockarie MJ Gatton ML Kemp DJ Good MF Anstey NM Gardiner DL Antibdy reactions to linear epitopes of *Plasmodium falciparum* cytoadherence-linked asexual gene 9 in asymptomatic children and adults from Papua New Guinea Am J Trop Med Hyg 2005 72 708 713 15964954 PMC2532502

[B42] Pain A Renauld H Berriman M Murphy L Yeats CA Weir W Kerhornou A Aslett M Bishop R Bouchier C Cochet M Coulson RM Cronin A de Villiers EP Fraser A Fosker N Gardner M Goble A Griffiths-Jones S Harris DE Katzer F Larke N Lord A Maser P McKellar S Mooney P Morton F Nene V O'Neil S Price C Genome of the host-cell transforming parasite *Theileria annulata* compared with T. Parva Science 2005 309 131 133 10.1126/science.1110418 15994557

[B43] Bakheit MA Ahmed JS Seitzer U Existence of splicing variants in homologues of *Theileria lestoquardi* clone-5 Gene’s transcripts in Theileria annulata and Theileria parva Ann N Y Acad Sci 2008 1149 212 213 10.1196/annals.1428.023 19120213

[B44] Fernandez-Becerra C Yamamoto MM Vëncio RZN Lacerda M Rosanas-Urgell A del Portillo HA *Plasmodium vivax* and the importance of the subtelomeric multigene vir superfamily Trends Parasitol 2009 25 44 51 10.1016/j.pt.2008.09.012 19036639

[B45] Epp C Li F Howitt CA Chookajorn T Deitsch KW Chromatin associated sense and antisense noncoding RNAs are transcribed from the var gene family of virulence genes of the malaria parasite *Plasmodium falciparum* RNA 2009 15 116 127 19037012 10.1261/rna.1080109 PMC2612763

[B46] Kyes SA Rowe JA Kriek N Newbold CI Rifins: A second family of clonally variant proteins expressed on the surface of red cells infected with *Plasmodium falciparum* Proc Natl Acad Sci U S A 1999 96 9333 9338 10.1073/pnas.96.16.9333 10430943 PMC17783

[B47] Niang M Yan Yam X Preiser PR The *Plasmodium falciparum* STEVOR multigene family mediates antigenic variation of the infected erythrocyte PLoS Pathog 2009 5 e1000307 10.1371/journal.ppat.1000307 19229319 PMC2637975

[B48] Blythe JE Surentheran T Preiser PR STEVORa multifunctional protein? Mol Biochem Parasit 2004 134 11 15 10.1016/j.molbiopara.2003.09.011 14747138

[B49] Sutherland CJ Stevor transcripts from *Plasmodium falciparum* gametocytes encode truncated polypeptides Mol Biochem Parasit 2001 113 331 335 10.1016/S0166-6851(01)00225-0 11295189

[B50] Sanyal S Egée S Bouyer G Perrot S Safeukui I Bischoff E Buffet P Deitsch KW Mercereau-Puijalon O David PH Templeton TJ Lavazec C *Plasmodium falciparum* STEVOR proteins impact erythrocyte mechanical properties Blood 2012 119 e1 e8 10.1182/blood-2011-08-370734 22106347 PMC3257022

[B51] Tibúrcio M Niang M Deplaine G Perrot S Bischoff E Ndour PA Silvestrini F Khattab A Milon G David PH Hardeman M Vernick KD Sauerwein RW Preiser PR Mercereau-Puijalon O Buffet P Alano P Lavazec C A switch in infected erythrocyte deformability at the maturation and blood circulation of *Plasmodium falciparum* transmission stages Blood 2012 119 e172 e180 10.1182/blood-2012-03-414557 22517905 PMC3382942

[B52] Dixit R Sharma A Mourya DT Kamaraju R Patole MS Shouche YS Salivary gland transcriptome analysis during *Plasmodium* infection in malaria vector *Anopheles stephensi* Int J Infect Dis 2009 13 636 646 10.1016/j.ijid.2008.07.027 19128996

[B53] Levy F Bulet P Ehret-Sabatier L Proteomic analysis of the systemic immune response of *Drosophila* Mol Cell Proteomics 2004 3 156 166 14645501 10.1074/mcp.M300114-MCP200

[B54] Grolli S Merli E Conti V Scaltriti E Ramoni R Odorant binding protein has the biochemical properties of a scavenger for 4-hydroxy-2-nonenal in mammalian nasal mucosa FEBS J 2006 273 5131 5142 10.1111/j.1742-4658.2006.05510.x 17042783

[B55] Sim S Ramirez JL Dimopoulos G Dengue virus infection of the *Aedes aegypt*i salivary gland and chemosensory apparatus induces genes that modulate infection and blood-feeding behavior PLoS Pathog 2012 8 e1002631 10.1371/journal.ppat.1002631 22479185 PMC3315490

[B56] Danielli A Kafatos FC Loukeris TG Cloning and characterization of four *Anopheles gambiae* serpin isoforms, differentially induced in the midgut by *Plasmodium berghe*i invasion J Biol Chem 2003 278 4184 4193 10.1074/jbc.M208187200 12456678

[B57] Christophides GK Zdobnov E Barillas-Mury C Birney E Blandin S Blass C Brey PT Collins FH Danielli A Dimopoulos G Hetru C Hoa NT Hoffmann JA Kanzok SM Letunic I Levashina EA Loukeris TG Lycett G Meister S Michel K Moita LF Müller HM Osta MA Paskewitz SM Reichhart JM Rzhetsky A Troxler L Vernick KD Vlachou D Volz J Immunity-related genes and gene families in *Anopheles gambiae* Science 2002 298 159 165 10.1126/science.1077136 12364793

[B58] Smith PH Mwangi JM Afrane YA Yan G Obbard DJ Ranford-Cartwright LC Little TJ Alternative splicing of the *Anopheles gambiae* Dscam gene in diverse *Plasmodium falciparum* infections Malaria J 2011 10 156 10.1186/1475-2875-10-156 PMC3118162 21651790

[B59] Robertson AS Belorgey D Lilley KS Lomas DA Gubb D Dafforn TR Characterization of the necrotic protein that regulates the toll-mediated immune response in *Drosophila* J Biol Chem 2003 278 6175 6180 10.1074/jbc.M209277200 12414799

[B60] The UniProt C Reorganizing the protein space at the universal protein resource (UniProt) Nucleic Acids Res 2012 40 D71 D75 22102590 10.1093/nar/gkr981 PMC3245120

[B61] Suwanchaichinda C Kanost MR The serpin gene family in Anopheles gambiae Gene 2009 442 47 54 10.1016/j.gene.2009.04.013 19394412 PMC2716094

[B62] Marygold SJ Leyland PC Seal RL Goodman JL Thurmond J Strelets VB Wilson RJ the FlyBase c FlyBase: improvements to the bibliography Nucleic Acids Res 2013 41 D751 D757 10.1093/nar/gks1024 23125371 PMC3531214

[B63] Choe K-M Lee H Anderson KV Drosophila peptidoglycan recognition protein LC (PGRP-LC) acts as a signal-transducing innate immune receptor Proc Natl Acad Sci U S A 2005 102 1122 1126 10.1073/pnas.0404952102 15657141 PMC545828

[B64] Meister S Agianian B Turlure F Relógio A Morlais I Kafatos FC Christophides GK *Anopheles gambiae* PGRPLC-mediated defense against bacteria modulates infections with malaria parasites PLoS Pathog 2009 5 e1000542 10.1371/journal.ppat.1000542 19662170 PMC2715215

[B65] Dong Y Taylor HE Dimopoulos G AgDscam, a hypervariable immunoglobulin domain-containing receptor of the *anopheles gambiae* innate immune system PLoS Biol 2006 4 e229 10.1371/journal.pbio.0040229 16774454 PMC1479700

[B66] Berriman M Haas BJ LoVerde PT Wilson RA Dillon GP Cerqueira GC Mashiyama ST Al-Lazikani B Andrade LF Ashton PD Aslett MA Bartholomeu DC Blandin G Caffrey CR Coghlan A Coulson R Day TA Delcher A DeMarco R Djikeng A Eyre T Gamble JA Ghedin E Gu Y Hertz-Fowler C Hirai H Hirai Y Houston R Ivens A Johnston DA The genome of the blood fluke *Schistosoma mansoni* Nature 2009 460 352 358 10.1038/nature08160 19606141 PMC2756445

[B67] DeMarco R Mathieson W Manuel SJ Dillon GP Curwen RS Ashton PD Ivens AC Berriman M Verjovski-Almeida S Wilson RA Protein variation in blood-dwelling schistosome worms generated by differential splicing of micro-exon gene transcripts Genome Res 2010 20 1112 1121 10.1101/gr.100099.109 20606017 PMC2909574

[B68] Davis RE Davis AH Carroll SM Rajkovic A Rottman FM Tandemly repeated exons encode 81-base repeats in multiple, developmentally regulated *Schistosoma manson*i transcripts Mol Cell Biol 1988 8 4745 4755 3211127 10.1128/mcb.8.11.4745-4755.1988 PMC365566

[B69] Almeida GT Amaral MS Beckedorff FCF Kitajima JOP DeMarco R Verjovski-Almeida S Exploring the *Schistosoma manson*i adult male transcriptome using RNA-seq Exp Parasitol 2010 132 22 31 21745473 10.1016/j.exppara.2011.06.010

[B70] Parker-Manuel SJ Ivens AC Dillon GP Wilson RA Gene expression patterns in larval *Schistosoma manson*i associated with infection of the mammalian host PLoS Negl Trop D 2011 5 e1274 10.1371/journal.pntd.0001274 PMC3166049 21912711

[B71] DeMarco R Oliveira KC Venancio TM Verjovski-Almeida S Gender biased differential alternative splicing patterns of the transcriptional cofactor CA150 gene in *Schistosoma mansoni* Mol Biochem Parasit 2006 150 123 131 10.1016/j.molbiopara.2006.07.002 16904200

[B72] Chalmers IW McArdle AJ Coulson RM Wagner MA Schmid R Hirai H Hoffmann KF Developmentally regulated expression, alternative splicing and distinct sub-groupings in members of the Schistosoma mansoni venom allergen-like (SmVAL) gene family BMC Genomics 2008 9 89 10.1186/1471-2164-9-89 18294395 PMC2270263

[B73] Chalmers IW Hoffmann KF Platyhelminth venom allergen-like (VAL) proteins: revealing structural diversity, class-specific features and biological associations across the phylum Parasitology 2012 139 1231 1245 10.1017/S0031182012000704 22717097 PMC3435950

[B74] Farias LP Rodrigues D Cunna V Rofatto HK Faquim-Mauro EL Leite LCC *Schistosoma manson*i venom allergen like proteins present differential allergic responses in a murine model of airway inflammation PLoS Negl Trop D 2012 6 e1510 10.1371/journal.pntd.0001510 PMC3274501 22347513

[B75] Roger E Grunau C Pierce RJ Hirai H Gourbal B Galinier R Emans RM Cesari IM Cosseau C Mitta G Controlled chaos of polymorphic mucins in a metazoan parasite (*Schistosoma mansoni*) interacting with its invertebrate host (*Biomphalaria glabrata*) PLoS Negl Trop D 2008 2 e330 10.1371/journal.pntd.0000330 PMC2576457 19002242

[B76] Mone Y Gourbal B Duval D Du Pasquier L Kieffer-Jaquinod S Mitta G A large repertoire of parasite epitopes matched by a large repertoire of host immune receptors in an invertebrate host/parasite model PLoS Negl Trop D 2010 4 e813 10.1371/journal.pntd.0000813 PMC2935394 20838648

[B77] Hanington PC Forys MA Dragoo JW Zhang S-M Adema CM Loker ES Role for a somatically diversified lectin in resistance of an invertebrate to parasite infection Proc Natl Acad Sci U S A 2010 107 21087 21092 10.1073/pnas.1011242107 21084634 PMC3000291

[B78] Seed JR Protozoa: Pathogenesis and Defenses 1996 4 Galveston: University of Texas Medical Branch 21413293

[B79] Pfaff AW Candolfi E Immune responses to protozoan parasites and its relevance to diagnosis in immunocompromised patients Eur J Protistol 2003 39 428 434 10.1078/0932-4739-00016

[B80] Lejon V Ngoyi DM Ilunga M Beelaert G Maes I Büscher P Fransen K Low specificities of HIV diagnostic tests caused by *Trypanosoma brucei* gambiense sleeping sickness J Clin Microbiol 2010 48 2836 2839 10.1128/JCM.00456-10 20573878 PMC2916589

[B81] Gasasira AF Dorsey G Kamya MR Havlir D Kiggundu M Rosenthal PJ Charlebois ED False-positive results of enzyme immunoassays for human immunodeficiency virus in patients with uncomplicated malaria J Clin Microbiol 2006 44 3021 3024 10.1128/JCM.02207-05 16891532 PMC1594619

[B82] Everett DB Baisely KJ McNerney R Hambleton I Chirwa T Ross DA Changalucha J Watson-Jones D Helmby H Dunne DW Mabey D Hayes RJ Association of Schistosomiasis with false-positive HIV test results in an African adolescent population J Clinl Microbiol 2010 48 1570 1577 10.1128/JCM.02264-09 PMC2863920 20181896

[B83] Orlov M Vaida F Finney OC Smith DM Talley AK Wang R Kappe SH Deng Q Schooley RT Duffy PE *P. falciparum* enhances HIV replication in an experimental malaria challenge system PLoS One 2012 7 26 10.1371/journal.pone.0039000 PMC3383717 22745697

[B84] Ayash-Rashkovsky M Chenine AL Steele LN Lee SJ Song R Ong H Rasmussen RA Hofmann-Lehmann R Else JG Augostini P McClure HM Secor WE Ruprecht RM Coinfection with Schistosoma mansoni reactivates viremia in rhesus macaques with chronic simian-human immunodeficiency virus clade C infection Infect Immun 2007 75 1751 1756 10.1128/IAI.01703-06 17283092 PMC1865689

[B85] Secor WE Shah A Mwinzi PMN Ndenga BA Watta CO Karanja DMS Increased density of human immunodeficiency virus type 1 coreceptors CCR5 and CXCR4 on the surfaces of CD4^+^ T cells and monocytes of patients with *Schistosoma manson*i infection Infect Immun 2003 71 6668 6671 10.1128/IAI.71.11.6668-6671.2003 14573694 PMC219584

[B86] Andreani G Celentano AM Solana ME Cazorla SI Malchiodi EL Martinez Peralta LA Dolcini GL Inhibition of HIV-1 replication in human monocyte-derived macrophages by parasite *Trypanosoma cruzi* PLoS ONE 2009 4 e8246 10.1371/journal.pone.0008246 20011521 PMC2788415

[B87] Alcaide P Fresno M The *Trypanosoma cruz*i membrane mucin AgC10 inhibits T cell activation and IL-2 transcription through L-selectin Int Immunol 2004 16 1365 1375 10.1093/intimm/dxh138 15314038

[B88] Ip JY Tong A Pan Q Topp JD Blencowe BJ Lynch KW Global analysis of alternative splicing during T-cell activation RNA 2007 13 563 572 10.1261/rna.457207 17307815 PMC1831861

[B89] Fukuhara K Okumura M Shiono H Inoue M Kadota Y Miyoshi S Matsuda H A study on CD45 isoform expression during T-cell development and selection events in the human thymus Human Immunol 2002 63 394 404 10.1016/S0198-8859(02)00379-8 11975983

[B90] McNeill L Cassady RL Sarkardei S Cooper JC Morgan G Alexander DR CD45 isoforms in T cell signalling and development Immunol Lett 2004 92 125 134 10.1016/j.imlet.2003.10.018 15081536

[B91] Antoine-Moussiaux N Cornet A Cornet F Glineur S Dermine M Desmecht D A Non-cytosolic protein of *Trypanosoma evansi* induces CD45-dependent lymphocyte death PLoS ONE 2009 4 e5728 10.1371/journal.pone.0005728 19478957 PMC2685979

[B92] Mahalingam M Pozniak A McManus TJ Senaldi G Vergani D Peakman M Abnormalities of CD45 isoform expression in HIV infection Clin Immunol Immunop 1996 81 210 214 10.1006/clin.1996.0178 8906753

[B93] Martin DL Weatherly DB Laucella SA Cabinian MA Crim MT Sullivan S Heiges M Craven SH Rosenberg CS Collins MH Sette A Postan M Tarleton RL CD8+ T-cell responses to *trypanosoma cruz*i Are highly focused on strain-variant *trans* sialidase epitopes PLoS Pathog 2006 2 e77 10.1371/journal.ppat.0020077 16879036 PMC1526708

[B94] Muiá RP Yu H Prescher JA Hellman U Chen X Bertozzi CR Campetella O Identification of glycoproteins targeted by *Trypanosoma cruzi* trans-sialidase, a virulence factor that disturbs lymphocyte glycosylation Glycobiology 2010 20 833 842 10.1093/glycob/cwq037 20354005 PMC2900898

[B95] Mucci J Risso MG Leguizamón MS Frasch ACC Campetella O The trans-sialidase from Trypanosoma cruzi triggers apoptosis by target cell sialylation Cell Microbiol 2006 8 1086 1095 10.1111/j.1462-5822.2006.00689.x 16819962

[B96] Illingworth J Butler NS Roetynck S Mwacharo J Pierce SK Bejon P Crompton PD Marsh K Ndungu FM Chronic exposure to *Plasmodium falciparum* is associated with phenotypic evidence of B and T cell exhaustion J Immunol 2013 190 1038 1047 10.4049/jimmunol.1202438 23264654 PMC3549224

[B97] Moir S Fauci AS B cells in HIV infection and disease Nat Rev Immunol 2009 9 235 245 10.1038/nri2524 19319142 PMC2779527

[B98] Hall SR Heffernan BM Thompson NT Rowan WC CD4^+^CD45RA^+^ and CD4^+^CD45RO^+^ T cells differ in their TCR-associated signaling responses Eur J Immunol 1999 29 2098 2106 10.1002/(SICI)1521-4141(199907)29:07<2098::AID-IMMU2098>3.0.CO;2-B 10427972

[B99] Mummidi S Ahuja SS McDaniel BL Ahuja SK The human CC chemokine receptor 5 (CCR5) gene. Multiple transcripts with 5′-end heterogeneity, dual promoter usage, and evidence for polymorphisms within the regulatory regions and noncoding exons J Biol Chem 1997 272 30662 30671 10.1074/jbc.272.49.30662 9388201

[B100] Bamshad MJ Mummidi S Gonzalez E Ahuja SS Dunn DM Watkins WS Wooding S Stone AC Jorde LB Weiss RB Ahuja SK A strong signature of balancing selection in the 5′ cis-regulatory region of CCR5 Proc Natl Acad Sci U S A 2002 99 10539 10544 10.1073/pnas.162046399 12149450 PMC124967

[B101] Pease JE Murphy PM Microbial corruption of the chemokine system: an expanding paradigm Semin Immunol 1998 10 169 178 10.1006/smim.1998.0129 9653043

[B102] Camargo JF Quinones MP Mummidi S Srinivas S Gaitan AA Begum K Jimenez F VanCompernolle S Unutmaz D Ahuja SS Ahuja SK CCR5 Expression levels influence NFAT translocation, IL-2 production, and subsequent signaling events during T lymphocyte activation J Immunol 2009 182 171 182 19109148 10.4049/jimmunol.182.1.171 PMC2937277

[B103] Medeiros GA Silvério JC Marino APMP Roffê E Vieira V Kroll-Palhares K Carvalho CE Silva AA Teixeira MM Lannes-Vieira J Treatment of chronically *Trypanosoma cruzi*-infected mice with a CCR1/CCR5 antagonist (Met-RANTES) results in amelioration of cardiac tissue damage Microbes Infect 2009 11 264 273 10.1016/j.micinf.2008.11.012 19100857

[B104] Lasagni L Francalanci M Annunziato F Lazzeri E Giannini S Cosmi L Sagrinati C Mazzinghi B Orlando C Maggi E Marra F Romagnani S Serio M Romagnani P An alternatively spliced variant of CXCR3 mediates the inhibition of endothelial cell growth induced by IP-10, Mig, and I-TAC, and acts as functional receptor for platelet factor 4 J Exp Med 2003 197 1537 1549 10.1084/jem.20021897 12782716 PMC2193908

[B105] Rosas LE Barbi J Lu B Fujiwara Y Gerard C Sanders VM Satoskar AR CXCR3^−/−^ Mice mount an efficient Th1 response but fail to control Leishmania major infection Eur J Immunol 2005 35 515 523 10.1002/eji.200425422 15668916

[B106] Shikani HJ Freeman BD Lisanti MP Weiss LM Tanowitz HB Desruisseaux MS Cerebral malaria: we have come a long way Am J Pathol 2012 181 1484 1492 10.1016/j.ajpath.2012.08.010 23021981 PMC3483536

[B107] Miu J Mitchell AJ Müller M Carter SL Manders PM McQuillan JA Saunders BM Ball HJ Lu B Campbell IL Hunt NH Chemokine gene expression during fatal murine cerebral malaria and protection due to CXCR3 deficiency J Immunol 2008 180 1217 1230 18178862 10.4049/jimmunol.180.2.1217

[B108] Campanella GSV Tager AM El Khoury JK Thomas SY Abrazinski TA Manice LA Colvin RA Luster AD Chemokine receptor CXCR3 and its ligands CXCL9 and CXCL10 are required for the development of murine cerebral malaria Proc Natl Acad Sci U S A 2008 105 4814 4819 10.1073/pnas.0801544105 18347328 PMC2290783

[B109] Li H Gang Z Yuling H Luokun X Jie X Hao L Li W Chunsong H Junyan L Mingshen J Youxin J Feili G Boquan J Jinquan T Different neurotropic pathogens elicit neurotoxic CCR9- or neurosupportive CXCR3-expressing microglia J Immunol 2006 177 3644 3656 16951324 10.4049/jimmunol.177.6.3644

[B110] Streit WJ Microglia and Alzheimer’s disease pathogenesis J Neurosci Res 2004 77 1 8 10.1002/jnr.20093 15197750

[B111] Rock RB Gekker G Hu S Sheng WS Cheeran M Lokensgard JR Peterson PK Role of microglia in central nervous system infections Clin Microbiol Rev 2004 17 942 964 10.1128/CMR.17.4.942-964.2004 15489356 PMC523558

